# Northern Ghana final-year nurses’ attitudes towards nursing and remaining post qualification

**DOI:** 10.4102/curationis.v41i1.1832

**Published:** 2018-07-11

**Authors:** Atuut Abugri, Mary-Ann Jarvis

**Affiliations:** 1 School of Nursing and Public Health, University of KwaZulu-Natal, South Africa

## Abstract

**Background:**

Recruitment and retention concerns nursing globally, including Ghana, as the country attempts to meet health demands. A link exists between nursing students’ attitudes towards nursing and decisions to enter, remain in or withdraw from the profession.

**Objectives:**

To describe northern Ghana final-year student nurses’ current attitudes towards nursing and remaining in nursing post qualification.

**Method:**

Non-experimental quantitative descriptive design used convenient sampling targeting final third-year student nurses (*n* = 80) studying towards a Diploma in Registered General Nursing in a northern Ghana college (*N* = 220). Data were gathered using the attitude dimension of a self-administered questionnaire, developed by Al-Omar.

**Results:**

The response rate was 87.5% (*n* = 70). Respondents were 20–30 years of age, more men and predominantly from urban areas. The mean attitude dimension score (range 10–50) was 35.41 (SD 4.03) with no skewness (0.37); mean of single-item question about intention to stay in nursing was 3.68 (SD 1.14) with negative skewness (-0.92). Male and urban respondents’ attitudes were more positive than those of female respondents. No association was found between attitude score and demographics or intention to stay in nursing, but significant association was found between gender and habitation and attitude categories. Medium positive correlation existed between intent to stay in nursing and attitude score. Pay, travel opportunities and nursing being a challenging career attracted unfavourable attitudes.

**Conclusion:**

Ghanaian male student nurses’ attitudes are non-typical of general stereotypes held of nurses and gender, suggesting increased recruitment of male nurses. Demographic variables hold a small amount of value in the development of attitudes in Ghanaian nurses.

## Introduction

Globally, the demand for nurses continues to outstrip the available supply (WHO 2016). Nursing shortages prove to be a global challenge, as an under-resourced healthcare workforce attempts to deal with health needs, ultimately negatively influencing goals of improving patient care and health systems (Patidar et al. 2011:176; WHO 2016). Addressing both negative and positive attitudes held by nurses towards the profession (Fillman 2015:252; Miligi & Selim 2014:198; Patidar et al. 2011:176) represents an important aspect of understanding the issue of the nursing shortage (Al Jarrah 2013:147; Miligi & Selim 2014:197).

### Literature review

The prevailing attitude of nurses and society towards nursing is one of negativity (Glerean et al. 2017; Patidar et al. 2011:176) and a pivotal factor in the enduring nursing shortage (Rezaei-Adaryani, Salsali & Mohammadi 2012:85). It not only affects the recruitment of newly qualified nurses to nursing, but it is also associated with a high turnover of qualified nurses, and the intention of nursing students to leave the profession (Rezaei-Adaryani et al. 2012:85). Despite the evidence of negativity, society’s positive perceptions of nursing as a career (inclusive of Ghana) (Fillman 2015:252) has included helping others, entering tertiary education, job security, mobility, prestige, career variety, with a variance in the acclaims of the salary (Anarfi, Quartey & Agyei 2010:11; Fillman 2015:252; Lievens et al. 2011:18; Wilkes, Cowin & Johnson 2015). In the face of the nursing shortage, the positive perceptions are often linked to the reasons for nursing being a chosen career and what attracts applicants (Rhodes, Morris & Lazenby 2011). Unfortunately, this level of attraction lacks translation into an adequate supply of individuals who are willing to be nurses (Liu et al. 2017:12).

Further to the attitude towards nursing is the added problem of retention (Liu et al. 2017:1; Mokoka, Ehlers & Oosthuizen 2011:4) Nurses withdraw from the workforce through career changes, discontinuance of nursing practice, family responsibility or retirement (Mokoka et al. 2011). Alternate career options that offer more flexibility and better working conditions continue to lure nurses from the workforce (Duffield et al. 2009:12). Not surprisingly, nurses using nursing as a career ‘stepping stone’ have shorter nursing careers, with turnovers resulting in the incurrence of financial costs and costs to care delivery (Duffield et al. 2009:12; Fillman 2015:251).

Amidst the negative and positive portrayals, the active and passive motivators and nursing shortages sits the need to fulfil healthcare demands and meet the Sustainable Development Goals (Liu et al. 2017:2; Zamanzadeh et al. 2013:222). Recruitment and retention is a frequent concern for both nursing and midwifery across continents (Liu et al. 2017).

As far as recruitment is concerned, Ghana is no exception. In recent years, there has been an influx of applicants entering the nursing profession from young adults between the ages of 18 and 35 years (Mwini-Nyaledzigbor et al. 2014:26), yet healthcare managers in Ghana experience a critical shortage of qualified nurses (Anarfi et al. 2010; Mwini-Nyaledzigbor et al. 2014:27). A possible reason is that after graduation from colleges, much sooner than expected, nurses continue to leave the shores of Ghana or move their locus of care to non-governmental organisations (NGOs) (Anarfi et al. 2010:3; Donkor & Andrews 2011:222; Lievens et al. 2011:24; Mwini-Nyaledzigbor et al. 2014:26). In as much as it appears satisfying for the Nurse and Midwives Council of Ghana to have a large register of trained nurses, the attitudes and perceptions of nursing students need to be considered (Al Jarrah 2013:147; Bolan & Grainger 2009:775). Literature indicates that healthcare professionals’ morale in public healthcare facilities in Ghana is low (Anarfi et al. 2010:14). These attitudes have links to students’ decisions to either enter or continue in the nursing profession or to withdraw from nursing programmes (Al Jarrah 2013:147; Bolan & Grainger 2009:775).

### Problem statement

Ghana aligns with global nursing trends of attrition that need to be addressed (Mwini-Nyaledzigbor et al. 2014). The healthcare system in Ghana faces a human resource crisis as healthcare managers experience a critical shortage of qualified nurses (Anarfi et al. 2010:4; Mwini-Nyaledzigbor et al. 2014:26; Pillinger 2011:7). Amidst this shortage, nurses continue to leave the profession or Ghana sooner than expected (Mwini-Nyaledzigbor et al. 2014:26; Pillinger 2011:7). The attitude of society, individuals, nurses and nursing students towards the nursing profession contributes to the undersupply of nurses (Patidar et al. 2011:176; Zamanzadeh et al. 2013:220). Ghana nursing managers require evidence to strategise recruitment and retention and thereby allow for the meeting of the country’s health needs.

### Purpose of the study

The purpose of the study was to describe northern Ghana final-year student nurses’ current attitudes towards nursing and remaining in nursing post qualification, such that information could be available to assist in the recruitment and retention processes of nurses in Ghana.

### Objective of the study

To describe northern Ghana final-year student nurses’ current attitudes towards nursing and remaining in nursing post qualification

#### Contribution to the field

Ghana nursing managers require evidence to strategise recruitment and retention and thereby allow for the meeting of health needs.

## Background

Global nursing shortages (WHO 2016) are set to increase further as nurses leave the profession (Liu et al. 2017:12; Uğur & Kocaman 2011:65; WHO 2016). The result is an imbalance in nurse supply and demand, which creates a serious workforce crisis, affecting the ability to fight disease and improve health (Liu et al. 2017:3; WHO 2016). Estimations predict that the global deficit of skilled healthcare professionals will be 15 million by 2030 (Liu et al. 2017:6). Looking at the United States alone, shortages are suggested to grow to more than 1 million nurses by the year 2020 (Littlejohn et al. 2012:3).

Numerous contributory factors to the undersupply of nurses in developed and developing countries have been given by different authors (Anarfi et al. 2010; Dywili, Bonner & O’Brien 2013:512; Littlejohn et al. 2012:23). These factors include the ageing nurse workforce, declining nurse enrolment because of other more attractive alternate career opportunities for school leavers, emigration (Anarfi et al. 2010; Dywili et al. 2013:512; Littlejohn et al. 2012:23), as well as the poor nursing image and working conditions that result in job dissatisfaction (Al Jarrah 2013:147; Anarfi et al. 2010:14; Patidar et al. 2011:176; Zarea et al. 2009:32).

Ghana is aligned with global nursing trends of attrition, resulting in critical shortages of nurses (Mwini-Nyaledzigbor et al. 2014), with the existence of just over 22 000 nurses representing a ratio of under 10 nurses to 10 000 population, despite an increase in nurse recruitment since 2003 (Pillinger 2011:11). In a study on the psychosocial factors influencing the perception and choice of nursing as a profession, a positive correlation was found between student nurses’ intention to travel abroad after graduation and their choice of nursing as a career (Mwini-Nyaledzigbor et al. 2014). This finding can be linked to the attitudes student nurses have towards the nursing profession, which is a significant aspect in the issue of the nursing shortage (Al Jarrah 2013:147; Miligi & Selim 2014:197). Positive attitudes towards the nursing profession contribute to nurses remaining in the profession (Miligi & Selim 2014:197).

### Key concepts

#### Attitude

Attitude is defined as ‘a predisposition or a tendency to respond positively or negatively towards a certain idea, object, person, or situation’ (http://www.businessdictionary.com/definition/attitude.html).

#### Student nurse

Ghana defines a student nurse as a student enrolled and registered with a college or a university to pursue a nursing programme leading to the award of a certificate to practise nursing (www.nmcgh.org). In the study a final third-year nursing student is enrolled in a nursing college in northern Ghana for the Diploma in Registered General Nursing (RGN).

## Research methodology

### Research site

The study was carried out in a nursing college in northern Ghana, which is one of the six nursing colleges. The annual intake averages 80 students within the age range of 18–35 years with a male to female ratio of 1:1.

### Study design and target population

A non-experimental quantitative descriptive design was adopted, which focused on the 80 final third-year student nurses studying towards a Diploma in RGN at a college in northern Ghana (*N* = 220).

### Sampling

A three-step sampling procedure was used. Firstly, the researcher conveniently sampled the college, as the researcher was familiar with the setting; secondly, purposeful selection of the third-year group of students as opposed to the first or second years was done as they were exiting their training and had completed all the theoretical training in the classroom and all the clinical training in the wards. Lastly, there was no sampling of the 80 third-year student nurses and all were invited to participate as they met the inclusion criteria of being registered as a student, in their final year, present in class during data collection and voluntarily consented to participate in the study.

### Research instrument

A self-administered questionnaire was used. The questionnaire consisted of two sections: Section A was composed of three questions on demographic data (age, gender and habitation), requiring a direct response, and section B consisted of 11 questions relating to attitudes towards nursing. Ten of the questions were from the ‘attitude dimension’ of the ‘Knowledge, attitude and retention questionnaire’ developed by Al-Omar (2004). In addition an 11th question, separate to the attitude questions measured attitude towards staying in nursing. All 11 questions required responses on a five-point Likert scale.

Al-Omar (2004:152) (*n* = 641) reported the reliability coefficient of the attitude dimension as 0.71 which yielded a total mean score for the attitude dimension as 3.39 (SD 0.60), showing construct validity and internal consistency of the instrument. In the original questionnaire, content validity was achieved through the review of relevant literature and by hospital administration specialists and experienced nurse practitioners followed by a pilot study (Al-Omar 2004:152). In this study, Donabedian’s quality of care model focused on structures and processes, the objectives and the literature provided content validity for the questionnaire.

#### Data analysis

Data that were collected on 06 August 2015 were entered into IBM SPSS v 23, reverse scored as indicated, cleaned and then analysed for descriptive statistics, measures of central tendency, associations, correlations and reliability of the scale. Missing data, which were minimal, were handled by excluding cases pairwise in order to include all available data. The authors of the scale did not provide categories for the dimension outcomes (Al-Omar 2004:154); hence, the researcher used a mean split (35.41, SD 4.03; range 28–44/50) to create two categories of ‘less favourable attitudes’ (summation score range 28–34) and ‘favourable attitudes’ (summation score range 35–44).

### Ethical considerations

The head of the selected Ghana nursing college provided gatekeeper permission and ethical clearance was obtained from the researcher’s study university (HSS/0816/015H). All ethical boundaries were maintained prior to and during the obtaining of data from the respondents with a strong emphasis on anonymity and volunteering. Following an explanation of the study and obtaining written consent, the questionnaire was administered to respondents at one sitting and by a research assistant to decrease any potential risk. Data were stored on the researcher’s computer, which was password locked.

## Results

A response rate of 87.5% (*n* = 70) was obtained.

### Demographic data

Results showed that the majority of respondents (*n* = 61, 89.71%) were in the 20–25 age group, and only seven (10.29%) were in the category 26–30 years. There were no respondents in the 31- to 35-years age group. There were more male (*n* = 38, 56.72%) than female respondents (*n* = 29, 43.28%) and the majority of respondents were from urban areas (*n* = 38, 58.46%). Significant difference in age groups, gender and habitation were tested using chi-square (χ^2^) with significance set at *p* ˂ 0.05. The results showed that there was only a significant difference in the age groups (*n* = 68, χ^2^ = 42.88, *p* = 0.000), and no significance in gender (*n* = 67, *p* = 0.272) or habitation (*n* = 65, *p* = 0.172), showing the sample to be representative of the select college for gender and habitation.

### Attitude responses

Examining the individual items in the attitude dimension, 82.6% (*n* = 57) agreed or strongly agreed that nursing is a very interesting job. Similarly, a large extent of agreement (*n* = 65, 94.21%) was recorded for respondents being comfortable with the idea of being a nurse and enjoying caring and being with people (*n* = 68, 98.55%), as well as finding it very fulfilling to see patients recover (*n* = 66, 98%). On a less favourable note, there was a majority (*n* = 56, 81.16%) agreement in nursing being a challenging career with unreasonable pay (*n* = 40, 57.14%). Respondents were less inclined to view nursing as having travel opportunities (*n* = 35, 51.43%) with 38 (55.9%) perceiving that they could find a job in nursing wherever they went, which aligns with the minority (*n* = 28, 40.0%) identifying nursing as a secure profession.

### Examining the demographic related differences in responses

The male students (*n* = 37, 97.4%) were more comfortable than the female students (*n* = 25, 86.2%) with the idea of being a nurse and showed greater enjoyment in caring and being with people (*n* = 38, 100% vs. *n* = 27, 93.1%). However, the female respondents (*n* = 15, 51.7%) showed stronger agreement in nursing being a secure profession compared to the male respondents (*n* = 11, 29%). The dissatisfaction with the pay was driven by the students from urban locations (*n* = 23, 60.5%) as compared to those from rural settings (*n* = 15, 55.5%). Nursing as ‘a job’ for women came under disagreement by most of the respondents (*n* = 64, 91.43%).

### Associations of demographics with attitudes

The skewness value for the attitude dimension was 0.37. However, the sample size indicated the use of non-parametric testing (Mann–Whitney *U* test) for associations of the demographic variables of age groups, gender and habitation with the attitude dimension (significant difference was set at *p* ≤ 0.05) (Pallant 2010:229). The Mann–Whitney *U* test revealed no significant difference between the attitude score and age groups (*U* = −1.60, *p* = 0.361), nor habitation (*U* = −1.60, *p* = 0.111) and near significance in gender (*U* = −1.93, *p* = 0.054).

However, examining the association between the demographics and the two categories of attitudes created by the researcher (less favourable and favourable), the near-significant difference between male and female respondents using the attitude dimension score became significant (*U* = −2.25, *p* = 0.024). It was also noted that there was a significant difference between the attitude categories of favourable, less favourable and habitation (*U* = −2.08, *p* = 0.037) (see Table 1).

**TABLE 1:** Demographics associated with attitude score and attitude categories (*n* = 70).

Variable	Total respondents *n* (%)	Mann–Whitney (*U*) to attitude score	*p*	Mann–Whitney (*U*) to attitude category	*p*
**Age group (*n* = 68)**	-	−1.60	0.361	−1.10	0.360
20–25 years 26–30 years	61 (89.71) 7 (10.29)	- -	- -	- -	- -
**Gender (*n* = 67)**	-	−1.93	0.054	−2.25	0.024*
Male Female	38 (56.72) 29 (43.28)	- -		- -	- -
**Habitation (*n* = 65)**	-	−1.60	0.111	−2.08	0.037*
Rural Urban	27 (41.54) 38 (58.46)	- -	- -	- -	- -

Note: Difference in age, gender and habitation were tested using Mann–Whitney *U* test.

* , *p*-value of significance was set at < 0.05.

### Intention to stay in nursing

Results for the single-item question examining intent to stay in nursing revealed that the majority of respondents (*n* = 44, 63.8%) intended to stay in nursing post qualification (male respondents *n* = 24, 63.1%; female respondents *n* = 18, 62%). Respondents from urban areas intending to stay in nursing were the majority (*n* = 26, 68.4%). Mann–Whitney *U* test showed no significant differences between the intention to stay in nursing and the demographic variables of age group (*U* = −1.51, *p* = 0.613), gender (*U* = −1.96, *p* = 0.335) or habitation (*U* = −1.01, *p* = 0.989).

### Correlations

Correlation analysis using Spearman’s rho (*r*) identified the strength of the linear relationship between the attitude scale, attitude groups and intent to stay in nursing. Using Cohen’s interpretation (Pallant 2010:134), there was a medium positive correlation between intent to stay in nursing and attitude score (*r* = 0.37, *n* = 66, *p* = 0.002). The strength of correlation remained medium when testing against the two categories of attitudes (*r* = 0.33, *n* = 69, *p* = 0.008).

### Reliability

Cronbach’s alpha of 0.6 was obtained for the attitudes dimension which falls a little below the normal minimum value of 0.7 (Pallant 2010:100). It shows a questionable internal consistency of the items on the attitude dimension and for reliability for the setting.

## Discussion

The respondents were not representative of the Ghanaian student population as there were no respondents over 30 years of age. A Ghana requirement for student selection into training is that the applicant falls within the age range of 18–35 years and in the study respondents were mainly (89.71%) within the age group of 20–25 years. Students over 30 years are not usually many in the colleges, which reflects the Ghanaian educational system where a student who had uninterrupted training from primary school at the acceptable age of 6 years enters and graduates from college by the age of 20 years (MOH-Ghana 2014).

Despite the 31-35-year age group not being represented, there was no significant difference between represented age groups and attitude towards nursing (*U* = 1.60; *p* = 0.361), and, therefore, the practice of a cut-off at 35 years for new recruits is suggestive of review, thereby increasing the eligible applicants for nursing training. Anarfi et al. (2010:24) suggested an increase in the number of nurses undergoing training to counter the exit of nurses from Ghana. The finding of no significance between age groups and attitude is contrary to the significance (*p* = 0.05) found by Miligi and Selim (2014:205), when examining Saudi Arabian nursing students’ attitudes towards the nursing profession, as students in the age group 20–24 years showed more positive attitudes than the younger (less than 20 years) and older (25 years or more) age groups.

There is an increase in the number of male students joining nursing in Ghana with a ratio of 60% female to 40% male students (Lievens et al. 2011:16), where nursing is perceived as a suitable profession for both men and women (Ofori 2007:24). Ghanaian perceptions are unlike the more general perception that nursing is a career choice for women (Ashkenazi et al. 2017:166; Buthelezi et al. 2015:4; Zamanzadeh et al. 2013:51). The respondents’ agreement that nursing is not predominantly for women reflects Ghana’s non-stigmatising approach in terms of societal views on gender and nursing and the close ratio of male to female nurses (Lievens et al. 2011:16). In other countries such as Canada and the United States, there is gender bias that favours women as society views nursing as a female profession with only 5% of nurses being men; a similar situation exists for male representation in England (10%) and Ireland (4%) (Zamanzadeh et al. 2013:49). However, in recent times a revived interest for nursing amongst Ghanaian women shows that about 80% of female students who complete senior high school will opt for nursing over any other profession (Addy 2015). Furthermore, the Ghana nursing recruitment policy requires an equal ratio of male to female students (MOH-Ghana 2014).

Variance exists in the literature regarding gender and attitudes to nursing. Some authors report differences in gender and attitude towards nursing (Fillman 2015:252; Zamanzadeh et al. 2013:223), while others disagree in there being a difference (Mullan & Harrison 2008:533). In this study, there was a near-significant difference (*U* = −1.93, *p* = 0.054) in gender and the attitude score and a clearly significant difference (*U* = −2.25, *p* = 0.024) when comparing attitude categories. In contradiction to other findings (Ashkenazi et al. 2017; Buthelezi et al. 2015:6; Mahran & Al-Nagshabandi 2012:19), male respondents had a greater inclination to be more comfortable with the idea of being a nurse, seeing nursing as an interesting profession, with greater expressed enjoyment of caring and a sense of fulfilment in seeing patients getting better.

It is perhaps these positive attitudes that contribute to male nurses’ job satisfaction. Ofori (2007:24), examining the ‘glass elevator’ phenomenon in male nurses in Ghana, identified a high level of job satisfaction. This holds significance in the recruitment of male students who evidently hold the values of the nursing profession. Evidence of greater levels of caring in the male nurses in the study is counter to descriptions of female nurses as more maternal and caring than male nurses (Mahran & Al-Nagshabandi 2012:19), while some authors offer a possibility that male nurses could be as caring as female nurses (McMurry 2011:24).

Despite the portrayal of the positive attitudes, the majority (*n* = 40, 57.14%), especially the male nurses (*n* = 28, 73.6%) perceived that the pay in nursing is unreasonable. This is reflective of the current underfunding of the healthcare system in Ghana, forcing nurses to leave the country soon after training, into care provision in NGOs, which offer higher salaries (Anarfi et al. 2010:17; Mwini-Nyaledzigbor et al. 2014:26; Pillinger 2011:7). Ghanaian men have greater financial responsibilities (Zamanzadeh et al. 2013:51), which possibly explains the level of discontent with the salaries, yet it is noted that Anarfi et al. (2010:11) identified that the motivation to join nursing by Ghanaian male students was driven by financial gains and prestige. In favour of retention and despite the salary issue, Ghanaian male nurses are perceived as loyal to the profession and not discriminated against for promotion (Ofori 2007:25). Recognition must be given in this setting to the fact that men found nursing to be a less secure profession and the loyal trends of the past could change.

In addition to the male respondents, the urban respondents (*n* = 23, 60.5%) drove the discontent with wages, which matches the findings by Antwi and Phillips (2012:2) in that the impact of wages on attrition was strongest in urban areas. Despite these findings, the urban students, in comparison to the rural students, saw greater job security and opportunities for universal employment, which is suggestive of their exposure to other nursing environments in the urban setting (Miligi & Selim 2014:206) and an opportunity to moonlight (Lievens et al. 2011:11). Healthcare facilities in rural areas of Ghana are poorly resourced and sparsely located (Anarfi et al. 2010:9; Lievens et al. 2011:10). Furthermore, the financial dissatisfaction is suggestive of a catalyst in urban settings for nurses to move to other employment. The majority (*n* = 38, 58.46%) of respondents were from the urban areas, which could influence their choice of work setting post qualification, favouring an urban environment, drawn by a lifestyle of greater opportunity as compared to rural areas (Anarfi et al. 2010:9; Lievens et al. 2011:10). This has the potential to pose difficulties in retaining nurses for the rural areas (Anarfi et al. 2010:9; Lievens et al. 2011:9).

Ghana has instituted incentive schemes such as a Deprived Area Incentive Allowance for persons working in districts designated as deprived; however, recruitment to rural areas remains a challenge (Anarfi et al.2010:9; Lievens et al. 2011:21). Perhaps there is further learning Ghana can glean from South Africa’s rollout of the rural allowance (Ditlopo et al. 2011:589). It is important that Ghana revisits the Deprived Area Incentive Allowance, with ‘tangible handsome allowances’ as opposed to verbal promises, as well as exploring the constraints of working in rural areas (Donkor & Andrews 2011:222; Lievens et al. 2011:21). In addition, a possibility exists to examine the opportunity in rural areas for job progression, as the majority of the respondents (*n* = 44, 63.8%), in particular the male nurses (*n* = 24, 63.1%), intended to stay in nursing post qualification despite the long-existing problem of low salary levels (Anarfi et al. 2010; Pillinger 2011:8). In the retention of male nurses it is concerning that Ghana could lose loyal caring male nurses to the drawcard of money. It is of interest to note that just over half of the respondents (*n* = 35, 51.43%) did not see nursing to have opportunities for world travel, with limited perceptions (*n* = 35, 51.43%) on universal job availability. However, Ghana has lost large numbers of its nurses to emigration, often the United Kingdom (Anarfi et al. 2010:17: Donkor & Andrews 2011:222), which aligns with society’s view of nursing offering job security (Fillman 2015:252) and a stable future (Zamanzadeh et al. 2013:223).

The positive attitudes displayed by the respondents towards the nursing profession are relevant (Al-Omar 2004:153) and could have a strong sway over the deterrent of the salary. Miligi and Selim (2014:197) stated that positive attitudes towards the nursing profession contribute to an increase in the number of nurses who are devoted and stay in their profession. The predictive significance of positive attitudes towards retention and the respondents’ view that nursing is without universal employment prospects fails to answer the question of ‘why the attrition to places off Ghana’s shores’ (Anarfi et al. 2010; Mwini-Nyaledzigbor et al. 2014:26). Maybe the answer lies in current nurses’ changing view to stay within the shores of Ghana or answers will sought in a focus group of a future study. A focus group will also allow for further unpacking of the perception by the majority (*n* = 56, 81.16%) that nursing is a challenging career and not a secure profession (*n* = 42, 60.0%).

In examining habitation and attitude categories of favourable and less favourable, a significant difference (*U* = −2.08, *p* = 0.037) was found, which aligns with the study by Patidar et al. (2011:182) (*p* = 0.039) and gives meaning to the findings by Miligi and Selim (2014:206). The study by Miligi and Selim (2014:206) revealed that respondents from more modern and urbanised regions had positive attitudes towards nursing, which is explained by the presence of several colleges of nursing in public and private universities and students more oriented about nursing (Miligi & Selim 2014:206). In the context of Ghana’s geographical terrain, in the rural areas, resource restriction for equipment as well as a smaller proportion of skilled professionals (Anarfi et al. 2010:9), the study’s findings of significantly less favourable attitudes of the nurses from rural locations together presents a challenge to Ghanaian policymakers. In an attempt to meet the demand for nurses, the rural context suggests recruitment of nurses with stronger clinical reasoning skills capable of flexibility and thinking creatively. It is unfortunate that the nurse in the rural setting is at risk of disparagingly receiving the label of ‘the village nurse’ (Lievens et al. 2011:27). This further strengthens the previously mentioned need for a drawcard to attract into the rural areas the urban nurses (particularly the male nurses) who displayed more positive attitudes and were greater in number.

The lack of association between age (*p* = 0.613), gender (*p* = 0.335) and habitation (*p* = 0.989) with intention to stay in nursing is contrary to a previous study by El-Jardali et al. (2009:7), where age (*p* = 0.001) and gender (*p* = 0.037) had a significant bearing on intent to stay in nursing. Wang et al. (2012:551) also found age to be significant (*p* < 0.001). The medium positive correlation between intent to stay in nursing and the attitude dimension of the study is consistent with Al-Omar’s (2004:155) findings that the more positive attitude towards nursing, the more likely that the student would stay in nursing.

## Limitations

The study was not representative of all age groups. Hawthorne effect (Brink, Van der Walt & Van Rensburg 2012:164) seen in negative skewness might have influenced data relating to the intention to stay in nursing given that the students were in their final year and desiring employment post completion.

## Recommendations

Recruitment and retention strategies with related policies should focus on recruiting more men, improving the attitudes of female and rural nurses and, specific to Ghana, possibly increasing the limit of the recruitment age.

Furthermore, Ghana-specific recommendations are the implementation of a rural allowance to draw urban male nurses into rural areas, development of an attitude scale specific for the context of lower income countries and repeating the study for Ghana. Run focus groups to identify perceived challenges posed by nurses in order to intercept and address these earlier in nurses’ training.

## Conclusion

Generally, respondents (*n* = 70), in particular students from urban areas and male students of the select college had favourable attitudes towards nursing. Ghanaian male student nurses’ attitudes are non-typical of general stereotypes held of nurses and gender. However, amongst northern Ghana final-year student nurses, aspects that require focus in developing favourable attitudes, which could contribute towards retention and prevent early exit of nurses, are salary and addressing the perception that nursing is a challenging career.

The demographic variables hold a small amount of value in the development of attitudes in Ghanaian nurses and perhaps the greater link in attitude development lies in nurses’ training. Knowledge acquired through training develops favourable attitudes towards nursing (Miligi & Selim 2014:206).
